# Reference Genes for Expression Studies in Hypoxia and Hyperglycemia Models in Human Umbilical Vein Endothelial Cells

**DOI:** 10.1534/g3.114.013102

**Published:** 2014-09-05

**Authors:** Sherin Bakhashab, Sahira Lary, Farid Ahmed, Hans-Juergen Schulten, Ayat Bashir, Fahad W. Ahmed, Abdulrahman L. Al-Malki, Hasan S. Jamal, Mamdooh A. Gari, Jolanta U. Weaver

**Affiliations:** * Institute of Cellular Medicine, Newcastle University, Newcastle NE2 4HH, United Kingdom; † Biochemistry Department, King Abdulaziz University, P.O. Box 80203, Jeddah 21589, Saudi Arabia; ‡ Center of Excellence in Genomic Medicine Research, King Abdulaziz University, P.O. Box 80216, Jeddah 21589, Saudi Arabia; § Department of Obstetrics and Gynaecology, Faculty of Medicine, King Abdulaziz University, P.O. Box 80215, Jeddah 21589, Saudi Arabia

**Keywords:** HUVEC, hyperglycemia, hypoxia, reference genes

## Abstract

Human umbilical vein endothelial cell (HUVEC)-based gene expression studies performed under hypoxia and/or hyperglycemia show huge potential for modeling endothelial cell response in cardiovascular disease and diabetes. However, such studies require reference genes that are stable across the whole range of experimental conditions. These reference genes have not been comprehensively defined to date. We applied human genome-wide microarrays and quantitative real-time PCR (qRT-PCR) on RNA obtained from primary HUVEC cultures that were incubated for 24 hr either in euglycemic or in hyperglycemic conditions and then subjected to short-term CoCl_2_-induced hypoxia for 1, 3, or 12 hr. Using whole-transcript arrays, we selected 10 commonly used reference genes with no significant expression variation across eight different conditions. These genes were ranked using NormFinder software according to their stability values. Consequently, five genes were selected for validation by qRT-PCR. These were ribosomal protein large P0 (*RPLP0*), transferrin receptor (*TFRC*), glyceraldehyde-3-phosphate dehydrogenase (*GAPDH*), β-glucuronidase (*GUSB*), and β-actin (*ACTB*). All five genes displayed stable expression under hyperglycemia. However, only *RPLP0* and *TFRC* genes were stable under hypoxia up to 12 hr. Under hyperglycemia combined with hypoxia up to 12 hr, the expression of *RPLP0*, *TFRC*, *GUSB*, and *ACTB* genes remained unchanged. Our findings strongly confirm that *RPLP0* and *TFRC* are the most suitable reference genes for HUVEC gene expression experiments subjected to hypoxia and/or hyperglycemia for the given experimental conditions. We provide further evidence that even commonly known references genes require experimental validation for all conditions involved.

Cardiovascular disease (CVD) remains the main cause of morbidity and mortality in patients with diabetes mellitus (DM) (Ziche *et al.* 1997; Nakamura *et al.* 2006). With the advent of new pharmacological agents for diabetes, active research to improve the clinical outcome of CVD in those patients is ongoing. Impairment of endothelial repair is recognized as an early event in the development of CVD; therefore, *in vitro* studies involving human umbilical vein endothelial cells (HUVEC) are important for studying molecular mechanisms involved in CVD and the effect of potential new therapeutic agents.

A cardinal feature of CVD, particularly myocardial infarction, is tissue hypoxia. In patients with diabetes there are several risk factors involved in acute myocardial infarction, of which hypoxia, hyperglycemia, and the combination of both are fundamental and thus require depth investigation. To study the molecular events in CVD and diabetes, *in vitro* endothelial models considering the relevant conditions and time intervals that reflect the clinical situations are crucial. For accurate and reliable quantitative real-time PCR (qRT-PCR) results, normalization of data with reference genes is necessary. Reference genes, otherwise known as housekeeping genes, have a stable expression level in the tissues or cells undergoing investigation or different experimental conditions (Dheda *et al.* 2004; Huggett *et al.* 2005). Genome-wide expression analyses are powerful tools for a comprehensive gene screen of virtually all annotated genes and simultaneously compare expression data between different samples. qRT-PCR is the technique of choice to validate gene expression data generated by microarray experiments (Mutch *et al.* 2001; Provenzano and Mocellin 2007). Therefore, pilot studies are frequently conducted to establish reference genes for varying *in vivo* and *in vitro* conditions.

In the event of acute myocardial infarction/acute ischemia, cell death takes place in as little as 20 min in some animal models, whereas complete necrosis of all at-risk myocardial cells requires at least 2 to 4 hr or more (Jennings and Ganote 1974; Thygesen *et al.* 2007). Therefore, hypoxic model systems using short-term cultures between 1 and 3 hr are of paramount importance to appropriately simulate the time frame of acute ischemia. To date, no reference genes have been established in HUVEC cultures under hypoxia and/or hyperglycemia. Thus, the aim of our study was to determine reference genes in HUVEC cultures mimicking clinical conditions.

## Materials and Methods

### HUVEC cultures

The study was approved by The Biomedical Ethics Unit, Faculty of Medicine, King Abdulaziz University (approval number 440-10) and NRES Committee North East-Sunderland, UK (approval number 12/NE/0044). Following informed consent, umbilical cords were collected from normal deliveries and maintained in conservation buffer consisting of 50 ml DPBS containing 200 U/ml penicillin, 200 ug/ml streptomycin, and 2.5 ug/ml fungizone (PAA Laboratories, Buckinghamshire, UK). HUVEC were harvested from four independent umbilical cords by collagenase digestion (Roche, Basel, Switzerland) according to methods described by Jaffe *et al.* (1973). All experiments were performed in passage 2 at 50% to 60% confluency. The microarray experiments were repeated three times using three independent HUVEC samples for all tested conditions. The qRT-PCR experiments were performed using four independent HUVEC, three of which were the same as used in the microarray experiments.

### Hypoxia studies

HUVEC were cultured under normoxia (21% O_2_) or chemically induced hypoxia for 1, 3, and 12 hr using a final concentration of 150 µM CoCl_2_ (Sigma-Aldrich, Dorset, UK) according to an established protocol (Sultana *et al.* 1999).

### Hyperglycemia studies

HUVEC were cultured either with a euglycemic glucose concentration of 5.5 mM (Sigma-Aldrich, Dorset, UK) as a control or with hyperglycemic glucose concentration of 16.5 mM for 24 hr to mimic diabetes.

### Combined hyperglycemia and hypoxia studies

**Figure 1:**
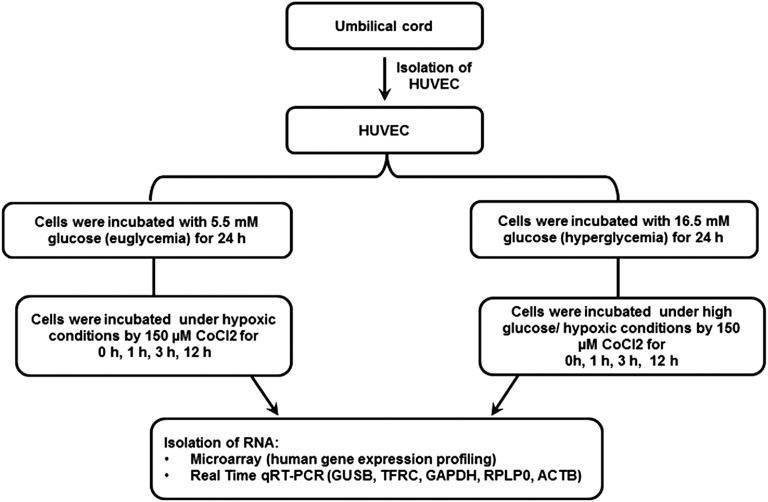
Experimental design. Human umbilical vein endothelial cells (HUVEC) from passage 2 were cultured either in medium containing euglycemic glucose concentration of 5.5 mM or in a hyperglycemic glucose concentration of 16.5 mM. After 24 hr, the euglycemic cultures and a portion of the hyperglycemic cultures were treated with 150 µM CoCl_2_ to induce chemical hypoxia. Then, RNA was extracted for gene expression analysis performed with microarrays and qRT-PCR.

HUVEC were cultured for 24 hr under hyperglycemic conditions and then hypoxia induced with CoCl_2_ for 1, 3, and 12 hr to mimic conditions during myocardial infarction in a diabetic patient. A summary of the experimental design is illustrated in Figure 1.

### Total RNA extraction

Total RNA from HUVEC was extracted using the RNeasy Mini Kit (QIAGEN, Hilden, Germany) according to the manufacturer’s instructions. On-column DNase digestion with RNase-Free DNase set (QIAGEN) was performed to remove genomic DNA contamination. The quality and quantity of RNA were assessed using a NanoDrop 2000c Spectrophotometer (Thermo Scientific, Wilmington, DE). The integrity of RNA samples was assessed by using Agilent 2100 Bioanalyzer (Santa Clara, CA).

### RNA sample preparation and microarray hybridization

Microarray experiments were performed using Affymetrix (Santa Clara, CA) Human Gene 1.0 ST arrays according to the manufacturer’s instructions with minor modifications (Merdad *et al.* 2014). For each sample, 250 ng of total RNA was processed using the Affymetrix GeneChip Whole Transcript Sense Target Labeling Assay. For the sample preparation, we used Ambion WT expression kit (Life technologies) and GeneChip WT Terminal Labeling and Controls Kit (Affymerix); 10,000 ng of cRNA was used for the second cycle cDNA reaction, and 5500 ng of the second cycle cDNA was fragmented and then labeled using GeneChip WT Terminal Labeling and Controls Kit. One µL of the fragmented and 1 µL of the fragmented and subsequently labeled cDNA were subjected to 3% agarose gel electrophoresis to determine fragmentation size, which usually ranged between 40 and 70 bp. Hybridization cocktails containing fragmented and end-labeled cDNA were applied to the Gene Chip Human Gene 1.0 ST arrays. Hybridization was performed for 17 ± 1 hr in the GeneChip Hybridization oven 640 at 45° and 60 rpm. Three different biological replicates were hybridized for each experimental condition, resulting in a total of 33 microarray experiments. Arrays were washed and stained using GeneChip Fluidics Station 450 FS450_007 Fluidics profile. The arrays were scanned using Affymetrix GeneChip scanner 3000 7G.

### Gene expression analysis

Affymetrix .CEL files were imported to Partek Genomics Suite version 6.6 (Partek Inc., St. Louis, MO). The data were normalized using robust multi-array average (RMA) normalization. Principal component analysis (PCA) was performed on all probes to visualize high dimensional data. A list of genes with gene expressions between −1.2-fold and 1.2-fold change was generated using ANOVA. Ten candidate reference genes that have been previously utilized in different studies under hypoxic conditions (Foldager *et al.* 2009; Bruge *et al.* 2011; Tan *et al.* 2012) were selected from our microarray data and then ranked by using NormFinder software (Andersen *et al.* 2004). NormFinder generated different stability values for a given set of candidate genes; a lower value implies higher stability in gene expression. The microarray data generated in this study were in compliance with MIAME (http://www.mged.org/Workgroups/MIAME/miame.html) guidelines. The complete dataset and associated experimental information have been submitted to NCBI’s Gene Expression Omnibus (GEO; http://www.ncbi.nlm.nih.gov/geo/) and are accessible through accession number GSE46263.

### Reverse-transcription reaction

Complementary DNA (cDNA) was synthesized using First-Strand cDNA Synthesis Kit (GE Healthcare, Little Chalfont, UK) according to the manufacturer’s guidelines. A solution of 20 µl RNA in RNase-free water with total RNA concentration of 500 ng was heated to 65° for 10 min. Subsequently, 11 µl of the bulk first-strand cDNA reaction mix, 1 µl of DTT solution, and 1 µl of random hexadeoxynucleotides primer were added to heat-denatured RNA. The samples were incubated for 1 hr at 37°.

### Real-time PCR

Primers were designed using the NCBI primer blast tool (http://www.ncbi.nlm.nih.gov/tools/primer-blast/) and were procured from Metabion (Steinkirchen, Germany). Primer sequences are listed in Table 1.

**Table 1:** PCR primers used in validation of gene expression by qRT-PCR

Gene-Specific Primers	Oligonucleotide Primer Sequence 5^′^ to 3^′^	Primer Length (bp)	Product Size (bp)	T_m_ (°C)
*RPLPO* Fw	CCATTCTATCATCAACGGGTACAA	24	75	62
Rw	TCAGCAAGTGGGAAGGTGTAATC	23		63
*GAPDH* Fw	GCCATCAATGACCCCTTCAT	20	81	58
Rw	GCCATGGAATTTGCCAT	17		50
*GUSB* Fw	CTACATCGATGACATCACCGTCAC	24	80	65
Rw	TGCCCTTGACAGAGATCTGGTAA	23		63
*TFRC* Fw	GTCGCTGGTCAGTTCGTGATT	21	80	61
Rw	AGCAGTTGGCTGTTGTACCTCTC	23		65
*ACTB* Fw	CCCTGGCACCCAGCAC	16	71	58
Rw	GCCGATCCACACGGAGTAC	19		62

The PCR mixture contained 1 µl of diluted cDNA, 10 µl of 2× Fast SYBR Green Master Mix (Life Technologies, Grand Island, NY), and 10 µM of each gene-specific primer in a total volume of 20 µl. Real-time reactions were performed in triplicates in 96-well optical reaction plates (Life Technologies) using StepOne Plus Real-Time PCR System (Life Technologies) under the following conditions: 20 sec at 95°, followed by 40 cycles of 3 sec at 95°, 30 sec at 60°, and 15 sec at 72°. Four different HUVECs were used for each condition.

### Statistical analysis

Mean expression values of each triplicate were calculated using the 2^−∆CT^ method, a modification of the 2^−∆∆CT^ method described earlier (Livak and Schmittgen 2001), wherein ∆C_t_ = C_t(sample)_ − C_t(control)_ and control is the sample without treatment. Expression values are presented as mean ± SEM. The comparisons of gene expression levels between qRT-PCR results were performed using paired *t*-test. Calculations were performed using SPSS software version 19.0 (SPSS Inc). *P* < 0.05 was considered statistically significant.

## Results

RNA quality analyses showed RNA integrity values (RIN) of 9.1–10. The algorithm assigns an RIN number score from 1 to 10, where level 10 represents completely intact RNA and 1 represents a highly degraded RNA.

### Expression levels of candidate reference genes by microarray

Microarray data (.CEL files) from three independent biological replicates were analyzed using Partek Genomics Suite version 6.6. Agglomerative average was performed and genes showing expressions between −1.2-fold and 1.2-fold change were considered stable. Therefore, a list of reference genes was generated by filtering genes that are stable under each condition (*P* > 0.05). We identified 9235 genes with no significant variation across all conditions. To narrow-down the number of candidate reference genes for all eight different conditions tested in our study, we selected 10 common reference genes to identify their stability values by using the NormFinder software (Table 2). Under hypoxia as well as under hyperglycemia and hyperglycemia combined with hypoxia for different time intervals (1, 3, and 12 hr), *RPLP0* was predicted to be the most stable expressed reference gene. Based on NormFinder analysis, we selected *RPLP0*, *ACTB*, *GAPDH*, *GUSB*, and *TFRC* for further qRT-PCR validation (Table 3).

**Table 2:** NormFinder microarray expression stability analysis

Gene Name	Hypoxia Stability Value	Hyperglycemia/Hypoxia Stability Value	Lower log2-Intensity	Upper log2-Intensity
***RPLP0***	0.008	0.004	13.05	13.31
***GAPDH***	0.008	0.005	13.81	14.05
***RPLP2***	0.009	0.005	13.03	13.18
***ACTB***	0.010	0.005	13.28	13.56
***HPRT1***	0.015	0.008	9.14	9.77
***GUSB***	0.016	0.008	8.92	9.65
***TFRC***	0.018	0.012	10.63	11.40
***ATP5F1***	0.019	0.010	7.20	7.94
***B2M***	0.020	0.012	10.44	11.58
***RPL13A***	0.023	0.028	9.86	11.18

Gene expression in the microarray was calculated by log2-intensity values of the genes. NormFinder transforms log to linear scale and ranks the candidate genes according to their expression stability, where a lower value indicates a higher stability in gene expression. The stability of genes was ranked into two groups, hypoxia for different time points (1, 3, 12 hr) and hyperglycemia/hyperglycemia combined with hypoxia for different time points (1, 3, 12 hr).

**Table 3:** Transcriptome analysis of five selected reference genes from HUVEC cultured under different conditions

Gene	*P* Hypoxia 1 hr	FC Hypoxia 1 hr	*P* Hypoxia 3 hr	FC Hypoxia 3 hr	*P* Hypoxia 12 hr	FC Hypoxia 12 hr	*P* High Glucose	FC High Glucose	*p* value High Glucose + Hypoxia 1 hr	FC High Glucose + Hypoxia 1 hr	*p* value High Glucose + Hypoxia 3 hr	FC High Glucose + Hypoxia 3 hr	*p* value High Glucose + Hypoxia 12 hr	FC High Glucose + Hypoxia 12 hr
***ACTB***	0.870	1.014	0.653	−1.039	0.154	1.132	0.428	1.070	0.320	1.089	0.519	1.056	0.343	1.085
***GAPDH***	0.962	1.002	0.877	−1.007	0.033	−1.101	0.410	−1.036	0.849	1.008	0.301	−1.046	0.051	−1.092
***GUSB***	0.993	−1.004	0.937	1.035	0.842	1.090	0.869	1.074	0.919	−1.044	0.926	1.041	0.879	−1.068
***RPLP0***	0.910	−1.006	0.731	−1.018	0.471	−1.038	0.535	−1.033	0.394	−1.045	0.416	−1.043	0.928	1.005
***TFRC***	0.836	1.049	0.890	−1.033	0.754	−1.076	0.997	1.001	0.828	−1.052	0.557	−1.147	0.886	1.034

Reference gene expressions from three independent microarray hybridizations were analyzed by Partek genomic suite version 6.6. Gene expressions for all conditions were compared against control sample, which is under euglycaemia and normoxia. Genes showing expression between −1.2-fold and 1.2-fold change and *P* > 0.05 were considered stable. FC, fold change; *ACTB*, actin beta; *GAPDH*, glyceraldehyde-3-phosphate dehydrogenase; *GUSB*, glucuronidase beta; *RPLP0*, ribosomal protein large P0; *TFRC*, transferrin receptor. The NCBI reference sequences (RefSeq) for: *ACTB*, NM_001101; *GAPDH*, NM_002046; *GUSB*, NM_000181; *RPLP0*, NM_001002; *TFRC*, NM_003234.

### *TFRC* and *RPLP0* mRNA expression were stable in HUVEC-induced by hypoxia

**Figure 2:**
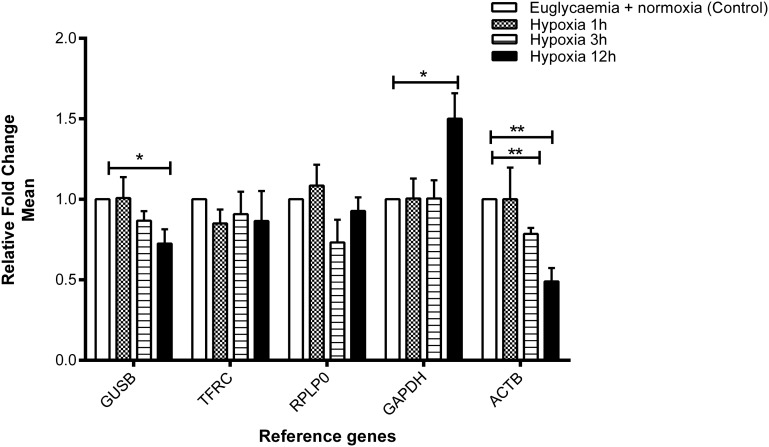
Effect of euglycemia and hypoxia on gene expression of the selected reference genes. Fold change in gene expression analyzed by the 2^−∆CT^ method. Data are mean ± SEM, n = 4, *P < 0.05, **P < 0.01 *vs.* control. Expression of *GUSB* and *GAPDH* was shown borderline significance after 12 hr of hypoxia, whereas *ACTB* was significantly changed after 3 and 12 hr of hypoxia. In contrast, the gene expression of *RPLP0* and *TFRC* was found to be stable compared with control.

Expression values of the five chosen reference genes were validated in four independent cDNA preparations. Under hypoxic conditions, the expression of *ACTB* was found to be stable after 1 hr of hypoxia compared with the control but significantly downregulated after 3 and 12 hr of hypoxia compared with the control (*P* = 0.01 and *P* = 0.009, respectively) (Figure 2). The expression of *GUSB* was found to be inhibited (*P* = 0.05), whereas *GAPDH* was upregulated with a borderline significance (*P* = 0.05) after 12 hr of hypoxia compared with the control (5.5 mM glucose). In contrast, *TFRC* and *RPLP0* displayed stable expression under hypoxia in all intervals (1, 3, and 12 hr) compared with the control.

### *GUSB*, *TFRC*, *RPLP0*, and *ACTB* mRNA expression were stable in HUVEC induced by hyperglycemia and hypoxia

**Figure 3:**
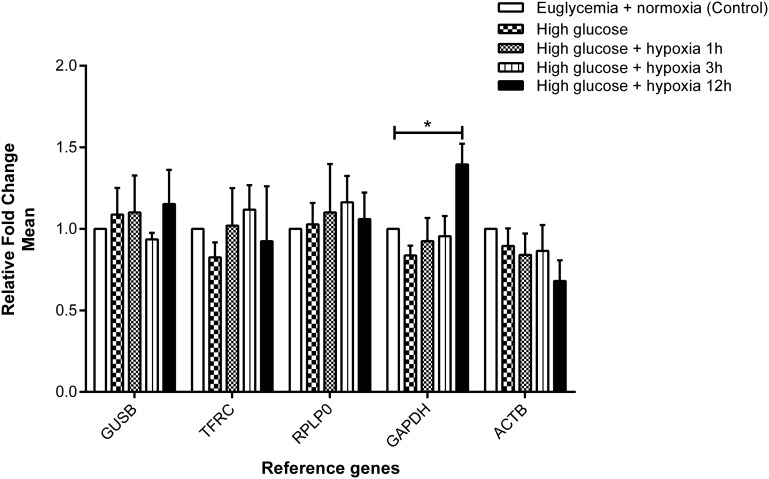
Effect of hyperglycemia and hypoxia on gene expression of the selected reference genes. Fold change in gene expression analyzed by the 2^−∆CT^ method. Data are mean ± SEM, n = 3–4, *P < 0.05 *vs.* control. Expression of *GUSB*, *TFRC*, *RPLP0*, and *ACTB* was found to be stable, whereas *GAPDH* was increased with borderline significance after 12 hr of hypoxia compared with the control.

When HUVEC were treated under hyperglycemic conditions (16.5 mM glucose) for 24 hr, the five selected reference genes demonstrated stable expression (Figure 3). Under hyperglycemia combined with hypoxia, gene expression of *GUSB*, *TFRC*, *RPLP0*, and *ACTB* turned out to be stable (Figure 3) in all time intervals measured (1, 3, and 12 hr). In contrast, an increase in gene expression of *GAPDH* was revealed with a borderline significance (*P* = 0.05) after 12 hr of hypoxic incubation compared with the control.

## Discussion

Better understanding of the cellular and molecular responses to hypoxia and hyperglycemia can lead to improved treatment of CVD in patients with diabetes. Myocardial infarction due to occlusion of one of the coronary arteries is frequently observed in such patients (Michiels 2004). Translational cardiovascular research *in vitro* requires an experimental design that reflects clinical conditions in patients to identify the affected genes.

In our *in vitro* studies, we investigated the conditions that are known to be associated with acute myocardial infarction under diabetic and nondiabetic conditions. We induced hypoxia using a hypoxia-mimetic agent, CoCl_2_. Earlier studies have shown that CoCl_2_ is able to generate a similar transcriptional response to reduced oxygen tension using hypoxia incubators (Vengellur *et al.* 2003; Poulios *et al.* 2006). The CoCl_2_ method has the advantage of being fast and inexpensive and provides a more stable hypoxia that is not possible with hypoxic chambers (Wu and Yotnda 2011). Our experiments show typical hypoxia response genes such as hypoxia-inducible factor-1α (*HIF1a*), vascular endothelial growth factor A (*VEGFA*), and glucose transporter 1 (*GLUT1*) to be overexpressed in hypoxia induced by CoCl_2_ (data submitted to GEO database), making our results comparable with low O_2_ tension experiments (Liu *et al.* 1999; Wang *et al.* 2007).

To mimic diabetic conditions, moderate hyperglycemia (16.5 mM glucose) was used. We avoided the commonly used excessive high glucose concentrations (22 mM) that have been shown to impair cellular growth in tissue culture. With 16.5 mM glucose concentration, no such effect was observed (Altannavch *et al.* 2004). A 16.5-mM glucose concentration not only is known to simulate a diabetic state but also is associated with hyperglycemia-mediated vascular inflammation through increased expression of endothelial adhesion molecules, such as endothelial-leukocyte adhesion molecule 1 (*ELAM-1*), vascular cell adhesion molecule-1 (*VCAM-1*), and intercellular adhesion molecule-1 (*ICAM-1*) (Takami *et al.* 1998; Altannavch *et al.* 2004). Our hyperglycemia experiments showed very high expression of thioredoxin interacting protein (*TXNIP*) in microarray results (data not shown). *TXNIP* has been recently shown to be a key mediator in hyperglycemia-mediated inflammatory response and beta-cell apoptosis (Minn *et al.* 2005; Zhou *et al.* 2010). Different exposure times to hypoxic and/or hyperglycemic conditions were selected to simulate both early and late cellular and molecular responses following ischemia (Thygesen *et al.* 2007). Of note, a qRT-PCR study of HUVEC cultured for 48 hr under different hypoxic conditions (1% O_2_, 5% CO_2_, with balanced N_2_, or 150 µM CoCl_2_) has demonstrated that human ATP synthase, H-transporting, mitochondrial F0 complex, subunit B1 (*ATP5F1*), *RPLP0*, and ribosomal protein large P2 (*RPLP2*) were suitable reference genes (Hatipoglu *et al.* 2009). In contrast, we applied shorter incubation times (1, 3, and 12 hr) and identified two reference genes, namely *RPLP0* and *TFRC*, as the most stably expressed under both euglycemia and hyperglycemia, in the presence or absence of hypoxia. In our results, two commonly perceived reference genes *GAPDH* and *ACTB* displayed significant differences in their expression levels under various hypoxic conditions. These results match an earlier study of bovine endothelial cells maintained in a humidified chamber flushed with 3% O_2_, where *GAPDH* showed unstable expression levels with a significant increase after 2–4 hr and a maximum level at 18 hr of hypoxia (Graven *et al.* 1994). From 9235 genes that were found stable in all studied conditions in microarray experiments, we analyzed 10 common reference genes using NormFinder. The NormFinder results showed *RPLP0* as the most stable reference gene that matches with our qRT-PCR data. However *GAPDH* and *ACTB* identified using the NormFinder as highly stable reference genes in hyperglycemia and hypoxia combined were not found in our validation by qRT-PCR. This difference in reference genes identified by microarray data using NormFinder and qRT-PCR could be due to nonconcordance of transcripts used in the microarray probe set and qRT-PCR (Dallas *et al.* 2005). The *TFRC* and *RPLP0* identified by us have been previously reported as reference genes in other cell types, including dexamethasone-exposed breast cancer cells and CD19-positive chronic lymphocytic leukemia cells (Abruzzo *et al.* 2005; Majidzadeh *et al.* 2011). Therefore, this is the first report indicating *TFRC* and *RPLP0* as suitable reference genes in HUVEC exposed to hypoxia, hyperglycemia, or hypoxia and hyperglycemia combined.

In summary, our data indicate not only that *TFRC* and *RPLP0* are suitable reference genes to normalize gene expression level in qRT-PCR experiments in hypoxic and/or hyperglycemic HUVEC cultures but also that commonly known reference genes always need to be validated for the given experimental conditions.
